# Numerical study of real gas effects during bubble collapse using a disequilibrium multiphase model

**DOI:** 10.1016/j.ultsonch.2022.106175

**Published:** 2022-10-01

**Authors:** Saeed Bidi, Phoevos Koukouvinis, Andreas Papoutsakis, Armand Shams, Manolis Gavaises

**Affiliations:** a School of Engineering and Mathematical Sciences, City University London, Northampton Square, EC1V 0HB London, UK; b Institut Jean le Rond d’Alembert, Sorbonne Université and CNRS UMR 7190, F-75005 Paris, France

**Keywords:** Bubble collapse, Tabulated EoS, Real gas data, Thermal effect

## Abstract

An explicit density-based solver of the Euler equations for inviscid and immiscible gas–liquid flow media is coupled with real-fluid thermodynamic equations of state supporting mild dissociation and calibrated with shock tube data up to 5000 K and 28 GPa. The present work expands the original 6-equation disequilibrium method by generalising the numerical approach required for estimating the equilibrium pressure in computational cells where both gas and liquid phases co-exist while enforcing energy conservation for all media. An iterative numerical procedure is suggested for taking into account the properties of the gas content as derived from highly non-linear real gas equations of state and implemented in a tabulated form during the numerical solution. The developed method is subsequently used to investigate gaseous bubble collapse cases considering both spherical and 2D asymmetric arrangements as induced by the presence of a rigid wall. It is demonstrated that the predicted maximum temperatures are strongly influenced by the equations of state used; the real gas model predicts a temperature reduction in the bubble interior up to 41% space-averaged and 50% locally during the collapse phase compared to the predictions obtained with the aid of the widely used ideal gas approximation.

## Highlights

• The ideal gas EoS is invalid for prediction of the bubble collapse temperature.
• Real gas EoSs capture the correct thermodynamics at high pressures.
• Real gas EoSs predict lower collapse temperature with 6000 K difference.
• 6-equation multiphase method is generalised for any arbitrary equation of state.

## Graphical abstract

**Figure:**
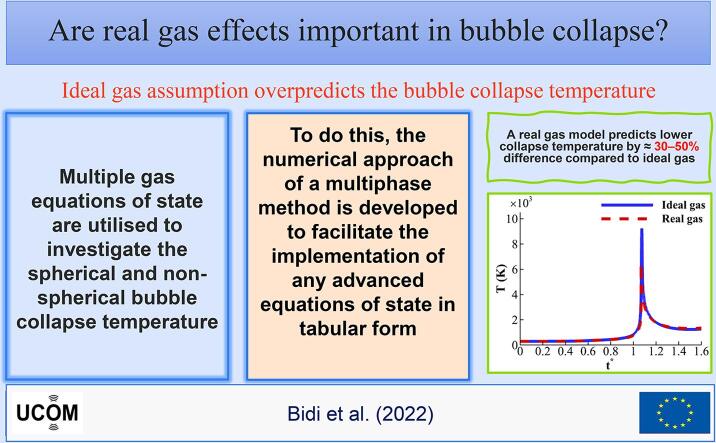

## Nomenclature

**Abbrevations**
BN: Baer-Nunziato
CFL: Courant-Friedrichs-Lewy number
DIM: Diffuse Interface Method
EoS: Equation of State
HLLC: Harten-Lax-van Leer-Contact
IG: Ideal gas
MUSCL: Monotonic Upstream-centered Scheme for Conservation Laws
PDE: Partial Differential Equation
PR: Peng-Robinson
RK: Redlich-Kwong
RKPR: Redlich-Kwong-Peng-Robinson
SG: Stiffened gas

**superscripts**
(a): After relaxation step
(b): Before relaxation step
∗: perturbated state
☆: updated value in Newton’s loops
n: time step

**Subscripts**
f: far-field
i: cell index in r-direction
j: cell index in z-direction
k: Phases’ index (k=1,2)
m: mixture
cons: conservative part

**List of symbols**
α: Volume fraction
β: Coordinate switch parameter
Δ: shifting value
∊: Residual
γ: Specific heat ratio
μ: Relaxation coefficient (m^2^·s/kg)
ϕ: Numerical Schlieren
ψ: Numerical Schlieren scaling parameter
ρ: Density (kg/m^3^)
F: r-direction flux vector
G: z-direction flux vector
$L_{R}$: Equations’ right hand side
q: State vector
$s_{g}(q)$: Geometric source terms
$s_{\mathrm{nc}}(q)$: non-conservative source terms
$s_{\mathrm{rlx}}$: Relaxation source terms
U: Solution vector
ε: Error in iterative loops
ξ: shifting coefficient
c: Speed of sound (m/s)
$c_{L}$: Reference speed of sound
$c_{\mathrm{urf}}$: Under-relaxation factor coefficient
E: Mixture total energy (J/kg)
e: Specific internal energy (J/kg)
$H_{0}$: Stand-off distance
J: Jacobian matrix
L: Domain size (m)
$L_{D}$: Domain size
N: Number of cells
p: Pressure (Pa)
$p_{\infty }$: Stiffened gas parameter
$p_{I}$: Interfacial pressure (Pa)
r: radial coordinate axis
$R^{*}$: non-dimensional radius
$R_{0}$: Initial bubble radius (m)
T: Temperature (K)
t: time
$t^{*}$: non-dimensional time
u: Velocity in r-direction (m/s)
v: Specific volume (m^3^/kg)
$V^{*}$: non-dimensional volume
w: Velocity in z-direction (m/s)
Y: Mass fraction
Z: Acoustic impedance (Pa·s/m^3^)
z: axial coordinate axis

## 1 Introduction

Thermal effects occurring during the collapse of gaseous bubbles [1], including sonoluminescence [2], air dissociation and chemical reactions [3] are now well documented. While the bulk liquid temperature does not change significantly compared to the inner bubble content [4], [5], [6], [7], the latter can reach enormous temperatures during the collapse, of the order of thousands of degrees Kelvin as computational studies for both spherical [8], [9] and non-spherical bubble collapse cases [10], [11] as well as molecular dynamics [12] suggest. A precise determination of the bubble thermodynamics is important in different areas such as in sonochemistry [13], [14] [1], [3] and ultrasound therapy such as High-intensity Focused Ultrasound (HIFU) to ensure safety and efficiency [15][4]. Numerical models utilised for the simulation of spherical bubble collapse under such extreme condition typically have employed zero-dimensional approximations, such as the Rayleigh-Plesset [16], Keller-Miksis [17], or Gilmore [18] models. Despite their simplicity and low computational cost, their simplifying assumptions such as spherical symmetry and spatial uniformity of the temperature, limit their applicability to describe the flow physics of more realistic configurations. These include, for example, the asymmetric bubbles collapse that occurs in presence of shocks or near solid boundaries. Moreover, homogeneity of the temperature distribution is affected by Peclet number. [19], [20].

To account for such effects and overcome the foregoing limitations of zero-dimensional models, multi-dimensional computational methods have been proposed. In this regard, there are different numerical approaches developed for predicting the temporal displacement of the gas–liquid interface, namely interface tracking [21], [22], [23], [24] or interface capturing [25], [26], [27]. Moreover, some developed approaches [28], [29], [30] are based on the solution of the zero-dimensional models. Regardless of the numerical approach, the thermodynamic closure utilised in the flow solvers plays a crucial role in predicting the temperature during the collapse. The vast majority of the relevant publications employ the ideal gas EoS (Equation of State); indicative studies include [31], [32], [33] for spherical and [34], [35], [36], [37], [38], [39], [40], [41] for non-spherical collapse cases.

Nevertheless, the ideal gas EoS does not provide accurate estimates of the gas temperature by ignoring ionization and dissociation as well as the dependency of the enthalpy on pressure which affects the compression heating or decompression cooling. There are only a few studies where a non-ideal gas EoS is used to model the thermodynamics of the bubble. Moss et al. [8] developed a 1D solver with spherical symmetry assumption and used an analytical EoS for the air bubble that includes vibrational excitation, dissociation, ionization, and a repulsive intermolecular potential. Extremely high temperatures up to $1.74\times 10^{6}$ K and $1.16\times 10^{7}$ K have been reported with and without considering the air dissociation and ionization respectively. In [9], [42] a 1D model of spherical bubble is employed where the vapour bubble thermodynamics was modelled as a hard-core van der Waals gas. It was shown that a 1 mm vapour bubble initially at saturation pressure surrounded by water at atmospheric pressure can reach a collapse temperature above $10^{4}$ K. The only work known to the authors that has considered non-ideal gas EoS in multi-dimensional flow solvers is [10]; a front tracking method was employed for resolving an argon bubble interface motion under the influence of a strong incident shock wave with varying pressure of 0.1–1000 GPa. The authors compared the results with ideal gas and two real gas EoSs namely the quotidian EoS, which utilise the thermodynamic functions from the Helmholtz free energy with the electronic contribution, and the SESAME database [43], [44], which considers the formation of plasma and ionization. It was revealed that the real gas EoSs estimate lower collapse temperatures depending on the shock strength; with a temperature difference of $3.5\times 10^{4}$ K and $1.5\times 10^{7}$ K for incident shock pressure of 1 and 1000 GPa, respectively.

In the present work we are using the six-equation method [45] to investigate thermal effects during bubble collapse accounting for the real gas thermodynamics. This method incorporates two distinct equations for the specific internal energy of each phase. Each phase is allowed to have different pressures. Mechanical equilibrium is imposed after the integration of the conservation equations, taking into account the conservation of the mixture’s total energy an equilibrium pressure is calculated. Here we present a numerical approach for imposing the mechanical equilibrium that can incorporate real gas EoSs in a tabulated form. Results are presented for three real gas EoS and compared against predictions obtained with the ideal gas EoS. To demonstrate the role of the non-ideal gas effects at different collapse strength, the spherical collapses are simulated at initial pressure ratios in the range of ≈ 7–353, defined as the initial liquid pressure over the gas pressure. Subsequently, a shock-induced non-spherical collapse in an ultrasound field of a lithotripter is simulated where the collapse pressure reaches the order of GPa.

The first aim of the present study is to gain insight into the impact of the real gas thermodynamics modeled by the cubic [46], [47] and Helmholtz [48] EoSs on an air bubble not only for a spherical collapse but also for a practical non-spherical case in Biomedical Science. For the latter, the wall pressure discussed in the case of using different gas EoSs. At the same time, the second aim is to develop the disequilibrium multiphase method [45] to be compatible for any arbitrary equation of state through tabulated data.

The remainder of this paper is organised as follows. The adopted equations of state are described in Section 2. The multiphase method and the adopted numerical schemes are explained in Section 3. The section presents various numerical results Section 4. Lastly, some concluding remarks are given in Section 5.

## 2 Equation of state

**Fig. 1:**
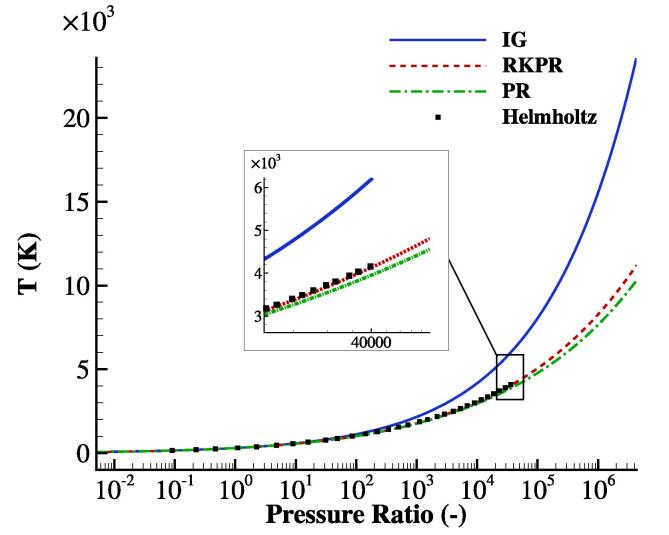
Comparison of the temperature obtained with ideal and real gas EoSs for different compression ratios in isentropic compression ($T_{0}=300$ K and $p_{0}=1$ bar).

In this study, the stiffened gas EoS [49] is employed for the liquid phase. For the gas phase, in addition to the ideal gas EoS, three distinct real gas EoSs are utilised, i.e.: (1) the Helmholtz EoS [48], and the cubic EoSs of: (2) Peng-Robinson (PR) [46] and (3) Redlich-Kwong Peng-Robinson (RKPR) [47]. Although the Helmholtz EoS is based on experimental data, its range of applicability does not cover the extreme thermodynamic state of the collapse studied in the current work. Therefore, herein, the cubic EoSs with the benefit of a wider range are also employed. The equations of state are described in A. To compare the behaviour of the gas EoSs, the deviations of temperature predictions among the equations of state for a simple isentropic gas compression are illustrated in Fig. 1 The compression starts from assumed values of 300 K and 1 bar for the temperature and pressure, respectively. It is clear that as the compression ratio (defined as the final over the initial pressure of 1 bar) increases, the difference between the ideal gas and real gas models (cubic and Helmholtz EoSs) increases. Notably, at a compression ratio of just 300, the difference in temperature prediction compared to that obtained using the ideal gas model is beyond 10%. At a compression ratio of $4\times 10^{4}$, this difference is higher than 35%. Such compression ratios are commonly found in bubble collapse cases. For example, in the study of a single bubble excited by a lithotripter [50], which is also simulated in the current work, the bubble starts the compression with the initial atmospheric pressure and reaches a collapse pressure more than 4 GPa. In another study [9], a laser induced bubble is initially at the vapour saturation pressure while it is compressed to the peak pressure of ≈10 GPa during the collapse. Comparing the three real gas models, it is observed that the RKPR EoS behaves practically identical to the Helmholtz EoS. The PR EoS, however, exhibits a ≈5% difference at a compression ratio of $4\times 10^{4}$ compared to the other two. Observing this, the RKPR EoS is preferred over the PR EoS for the non-spherical collapse case in this study where the bubble pressure reaches pressures higher than 4 GPa. It is noted in Fig. 1 that at higher compression ratios, comparison between the high order EoSs is not attempted, as the Helmholtz EoS is not reliable beyond its calibration range, giving unrealistic density variation as well as problematic values for heat capacity and speed of sound. This is a direct consequence of the high order nature of the Helmholtz model, which implies that its monotonicity is not guaranteed beyond the calibration range.

In this study, the stiffened gas and ideal gas EoSs are used in their parametric form due to the simplicity of their mathematical expressions. The real gas models, however, are implemented through tabular form. This offers a more versatile framework applicable to any EoS, i.e., the CFD solver does not need to be modified when using different tabulated EoSs. Moreover, it disengages the computational problem of evaluating the EoS from the CFD computations and circumnavigates the problem of deriving explicit solutions from the EoS with implicit expressions.

Each table of the real gas EoSs is a rectangular structured temperature - pressure grid with fixed intervals of T and $\log_{10}$ p. The considered range for temperature with 121 cells is [60,17000] K for the cubic and is [150,17000] K for the Helmholtz EoSs. The pressure with 375 cells ranges in $\left[2300,1.1\times 10^{10}\right]$ Pa for the cubic and $\left[2300,4\times 10^{9}\right]$ Pa for the Helmholtz EoSs. It will be shown that while the properties range for the cubic EoSs covers all the collapse cases in the present study, the Helmholtz EoS fails in simulation of the highest initial pressure ratio case.

## 3 Numerical method

In the present work, the bubble collapse is modelled using a so-called ‘six-equation model’ of [45] which stems from the Baer-Nunziato (BN) model [51]. In the BN model individual momentum and specific energy equations are considered for each phase. Thus, each phase possesses its own velocity, temperature and pressure, i.e., full disequilibrium. The BN model can be further simplified to reduced models [52], [53], that consider single velocity (kinetic equilibrium) and single pressure (mechanical equilibrium). However, these further reduced models present issues regarding volume fraction positivity and speed of sound monotonicity along with derivation difficulties for the Riemann solver when considering both phases according to [45]. To overcome these issues, the six-equation model of [45] was developed in which the phases have the same velocity but different pressures and temperatures. According to its developers [45], the six-equation model is not a physical model but a step model for the reduced models to overcome the mentioned issues of [52]. In this model, the phases will reach the mechanical equilibrium through a relaxation process at infinite rate which will be described in the solution algorithm. The model incorporates the mass and the energy conservation equations for each phase, a single momentum conservation equation for the mixture (considered as one equation in vector form) and the volume fraction transport equation for the first phase. In addition, the mixture total energy conservation equation is also solved as the seventh equation to ensure the total energy conservation. More details about this model can be found in literature, e.g., [54], [31], [33], [55], [56].

In the present work, the six-equation model is used in the 1D spherical and 2D axisymmetric coordinates (cylindrical coordinates with azimuthal symmetry) to save computational cost:

(1) $$\frac{\partial q}{\partial t}+\frac{\partial F}{\partial r}+\frac{\partial G}{\partial z}=s_{\mathrm{rlx}}+s_{\mathrm{nc}}\left(q\right)+s_{g}\left(q\right),$$

where:

$$\begin{array}{cc} q=\left[\begin{array}{c} \alpha_{1}\\ \alpha_{1}\rho_{1}\\ \alpha_{2}\rho_{2}\\ \rho u\\ \rho w\\ \rho E\\ \alpha_{1}\rho_{1}e_{1}\\ \alpha_{2}\rho_{2}e_{2} \end{array}\right],\; & F=\left[\begin{array}{c} \alpha_{1}u\\ \alpha_{1}\rho_{1}u\\ \alpha_{2}\rho_{2}u\\ \rho u^{2}+(\alpha_{1}p_{1}+\alpha_{2}p_{2})\\ \rho \mathrm{uw}\\ \left(\rho E+(\alpha_{1}p_{1}+\alpha_{2}p_{2})\right)u\\ \alpha_{1}\rho_{1}e_{1}u\\ \alpha_{2}\rho_{2}e_{2}u \end{array}\right],\\ G=\left[\begin{array}{c} \alpha_{1}w\\ \alpha_{1}\rho_{1}w\\ \alpha_{2}\rho_{2}w\\ \rho \mathrm{uw}\\ \rho w^{2}+(\alpha_{1}p_{1}+\alpha_{2}p_{2})\\ \left(\rho E+(\alpha_{1}p_{1}+\alpha_{2}p_{2})\right)w\\ \alpha_{1}\rho_{1}e_{1}w\\ \alpha_{2}\rho_{2}e_{2}w \end{array}\right],\; & s_{\mathrm{rlx}}=\left[\begin{array}{c} \mu \left(p_{1}-p_{2}\right)\\ 0\\ 0\\ 0\\ 0\\ 0\\ -p_{I}\mu \left(p_{1}-p_{2}\right)\\ p_{I}\mu \left(p_{1}-p_{2}\right) \end{array}\right],\\ s_{\mathrm{nc}}=\left[\begin{array}{c} -\alpha_{1}(\frac{\partial u}{\partial r}+\frac{\partial w}{\partial z})\\ 0\\ 0\\ 0\\ 0\\ 0\\ \alpha_{1}p_{1}(\frac{\partial u}{\partial r}+\frac{\partial w}{\partial z})\\ \alpha_{2}p_{2}(\frac{\partial u}{\partial r}+\frac{\partial w}{\partial z}) \end{array}\right],\; & s_{g}=-\frac{\beta }{r}\left[\begin{array}{c} 0\\ \alpha_{1}\rho_{1}u\\ \alpha_{2}\rho_{2}u\\ \rho u^{2}\\ \rho \mathrm{uw}\\ u\left(\rho E+(\alpha_{1}p_{1}+\alpha_{2}p_{2})\right)\\ u\left(\alpha_{1}\rho_{1}e_{1}+\alpha_{1}p_{1}\right)\\ u\left(\alpha_{2}\rho_{2}e_{2}+\alpha_{2}p_{2}\right) \end{array}\right], \end{array}$$

where $s_{\mathrm{rlx}},s_{\mathrm{nc}}$, and $s_{g}$ indicate relaxation, non-conservative, and geometric source terms, respectively. Moreover, subscripts 1 and 2 denote the water and air phases, respectively. Also, the following notation is adopted: r,z (coordinate axes), *t* (time), ρ (density), *p* (pressure), α (volume fraction), *u* (*r*-direction velocity), *w* (*z*-direction velocity), *e* (specific internal energy), *E* (specific total energy). The interfacial pressure $p_{I}$ is defined as follows which is an estimate in the limit of equal velocities introduced first in [57]:

(2) $$p_{I}=\frac{Z_{2}p_{1}+Z_{1}p_{2}}{Z_{1}+Z_{2}},$$

where $Z_{k}=\rho_{k}c_{k}$ denotes the acoustic impedance for phase *k* with speed of sound $c_{k}$. The value of 2 and 1 for the coordinates switching parameter β correspond to the 1D spherical the *r*-direction and 2D axisymmetric coordinates in the (r,z) directions, respectively. As can be seen in Eq. 2, this model assumes an initial disequilibrium pressure meaning that each phase has its own pressure. As pressure waves reach an interface, they propagate to the next phase by a pressure coupling between the phases. This coupling is controlled by the pressure equilibrium, characterised by very small time scales. The relaxation parameter μ quantifies the mechanical equilibrium time scale. The source terms in the specific internal energy equations appear to represent the exchange of the specific internal energies due to the pressure work.

The mixture speed of sound in this model is computed from:

(3) $$c_{m}^{2}=Y_{1}c_{1}^{2}+Y_{2}c_{2}^{2},$$

where *Y* denotes the mass fraction. As Eq. 1 denotes, the effects of viscosity, heat conductivity, surface tension, and phase transition are neglected in the present study. which presents a monotonic variation with volume fraction [45]. The numerical solution of the Eq. 1 is obtained through three major steps at each temporal loop:

1. Solving the hyperbolic part of the system. In this step, the pressure disequilibrium is assumed and the relaxation terms are ignored. This treatment gives a hyperbolic system for the conservative variables that is solved using an approximate Riemann solver with a finite volume scheme.
2. Converging to an equilibrium pressure by solving the relaxation system described in B.
3. Correcting of the solution by enforcing the total energy conservation.

### 3.1 Hyperbolic step

Considering the homogenous part of the PDEs in this step, Eq. 1 is solved using a finite volume Godunov method [58] with the second-order MUSCL scheme [59] employed to reconstruct the primitive variables at the cell boundary. Moreover, the HLLC approximate solver [60] is adopted to solve the Riemann problem at each cell boundary as an appropriate choice for the present method [45], [61], [55].

Using the computed inter-cell fluxes, the solution of the conservative and non-conservative variables can be evolved on the entire time step. The conventional Godunov scheme to update the conservative part of the system reads:

(4) $$\begin{array}{c} U_{i,j}^{n+1}=U_{i,j}^{n}-\frac{\Delta t}{\Delta r}\left[F_{\mathrm{cons}}^{*}\left(U_{i,j}^{n},U_{i+1,j}^{n}\right)-F_{\mathrm{cons}}^{*}\left(U_{i-1,j}^{n},U_{i,j}^{n}\right)\right]\\ -\frac{\Delta t}{\Delta z}\left[G_{\mathrm{cons}}^{*}\left(U_{i,j}^{n},U_{i,j+1}^{n}\right)-G_{\mathrm{cons}}^{*}\left(U_{i,j-1}^{n},U_{i,j}^{n}\right)\right]\\ +\Delta ts_{g,\mathrm{cons}}, \end{array}$$

in which:

$$\begin{array}{c} U={\left[\begin{array}{c} \alpha_{1}\rho_{1}\\ \alpha_{2}\rho_{2}\\ \rho u\\ \rho w\\ \rho E \end{array}\right]}^{T},F_{\mathrm{cons}}={\left[\begin{array}{c} \alpha_{1}\rho_{1}u\\ \alpha_{2}\rho_{2}u\\ \rho u^{2}+(\alpha_{1}p_{1}+\alpha_{2}p_{2})\\ \rho \mathrm{uw}\\ \left(\rho E+(\alpha_{1}p_{1}+\alpha_{2}p_{2})\right)u \end{array}\right]}^{T},\\ G_{\mathrm{cons}}={\left[\begin{array}{c} \alpha_{1}\rho_{1}w\\ \alpha_{2}\rho_{2}w\\ \rho \mathrm{uw}\\ \rho w^{2}+(\alpha_{1}p_{1}+\alpha_{2}p_{2})\\ \left(\rho E+(\alpha_{1}p_{1}+\alpha_{2}p_{2})\right)w \end{array}\right]}^{T},s_{g,\mathrm{cons}}=-\frac{\beta }{r}{\left[\begin{array}{c} \alpha_{1}\rho_{1}u\\ \alpha_{2}\rho_{2}u\\ \rho u^{2}\\ \rho \mathrm{uw}\\ \left(\rho E+(\alpha_{1}p_{1}+\alpha_{2}p_{2})\right)u \end{array}\right]}^{T}\text{.} \end{array}$$

The subscripts (i,j) stands for the finite volume cell index in (r,z) direction and superscript *n* shows the time step. Superscript ’*’ denotes the perturbated state. Calculation of $F_{\mathrm{cons}}^{*}$ and $G_{\mathrm{cons}}^{*}$ using the HLLC Riemann solver is explained in [45] and provided in C of the present work. The non-conservative part of the equations is updated following by approximating the volume integral with a midpoint rule and the divergences with a centred scheme [45]. Using this approximation, the volume fraction is updated as:

(5) $$\begin{array}{c} \alpha_{i,j}^{n+1}=\alpha_{i,j}^{n}-\frac{\Delta t}{\Delta r}\left[{\left(u\alpha \right)}_{i+\frac{1}{2},j}^{*}-{\left(u\alpha \right)}_{i-\frac{1}{2},j}^{*}-\alpha_{i,j}^{n}\left(u_{i+\frac{1}{2},j}^{*}-u_{i-\frac{1}{2},j}^{*}\right)\right]\\ -\frac{\Delta t}{\Delta z}\left[{\left(w\alpha \right)}_{i,j+\frac{1}{2}}^{*}-{\left(w\alpha \right)}_{i,j-\frac{1}{2}}^{*}-\alpha_{i,j}^{n}\left(w_{i,j+\frac{1}{2}}^{*}-w_{i,j-\frac{1}{2}}^{*}\right)\right], \end{array}$$

Assuming that the product ${\left(\alpha p\right)}_{i,j}^{n}$ is constant during the time step, the non-conservative internal energy equations can be approximated as [45]:

(6) $$\begin{array}{c} {\left(\alpha \rho e\right)}_{i,j}^{n+1}={\left(\alpha \rho e\right)}_{i,j}^{n}\\ -\frac{\Delta t}{\Delta r}\left[{\left(\alpha \rho \mathrm{eu}\right)}_{i+\frac{1}{2},j}^{*}-{\left(\alpha \rho \mathrm{eu}\right)}_{i-\frac{1}{2},j}^{*}-{\left(\alpha p\right)}_{i,j}^{n}\left(u_{i+\frac{1}{2},j}^{*}-u_{i-\frac{1}{2},j}^{*}\right)\right]\\ -\frac{\Delta t}{\Delta z}\left[{\left(\alpha \rho \mathrm{ew}\right)}_{i,j+\frac{1}{2}}^{*}-{\left(\alpha \rho \mathrm{ew}\right)}_{i,j-\frac{1}{2}}^{*}-{\left(\alpha p\right)}_{i,j}^{n}\left(w_{i,j+\frac{1}{2}}^{*}-w_{i,j-\frac{1}{2}}^{*}\right)\right], \end{array}$$

The approximation of the internal energy at this step is not crucial as it is used only to estimate the phasic pressures which will be corrected later in the relaxation step [45]. Finally, the solution is advanced using a two-step time integration [32]:

(7) $$q^{n+\frac{1}{2}}=q^{n}+\frac{1}{2}\Delta tL_{R}\left(q^{n}\right),$$

(8) $$q^{n+1}=q^{n}+\Delta tL_{R}\left(q^{n+\frac{1}{2}}\right),$$

where $L_{R}$ contains the right hand side of Eqs. (4), (5), (6).

### 3.2 Relaxation step

The hyperbolic step leads to different phasic pressures whereas the numerical solution should converge to a unique pressure to fulfil the mechanical equilibrium in the interface. This is achieved through the relaxation step. The concept behind the relaxation approach stems from two observations: firstly, when a rarefaction or shock wave passes through two phases having different pressures, the volume of each phase must change in order that pressures tend to equilibrium. The first relaxation term in $s_{\mathrm{rlx}}$ of Eq. 1 represents this volume fraction expansion with rate μ. Secondly, a pressure work is associated with the volume change of the phases. This is reflected by the last two relaxation terms in $s_{\mathrm{rlx}}$ of Eq. 1. Physically, μ depends on the mechanical properties of the fluids as well as the mixture topology [62], [63]. The stiff pressure relaxation with μ→∞ results in instantaneous pressure equilibrium at the interface at any time [62], [36]. In [45], [56], it is demonstrated the instantaneous pressure equilibrium is a valid assumption.

From the relaxation system described in B, the following equations can be derived:

(9) $$\frac{\partial e_{1}}{\partial t}+p_{I}\frac{\partial v_{1}}{\partial t}=0,$$

(10) $$\frac{\partial e_{2}}{\partial t}+p_{I}\frac{\partial v_{2}}{\partial t}=0,$$

in which $v_{k}=\frac{1}{\rho_{k}}$ shows the specific volume of phase *k*. The interfacial pressures $p_{I}$ in both phases are considered to be equal such that the mixture energy is conserved. A possible estimation of $p_{I}$ is the mixture pressure computed after the relaxation step [45], [56], which fulfils the entropy inequality (more details can be found in [64]). This allows the construction of a non-linear algebraic system:

(11) $$e_{1}^{\left(a\right)}-e_{1}^{\left(b\right)}-p^{\left(a\right)}\left(\frac{1}{\rho_{1}^{\left(a\right)}}-\frac{1}{\rho_{1}^{\left(b\right)}}\right)=0,$$

(12) $$e_{2}^{\left(a\right)}-e_{2}^{\left(b\right)}-p^{\left(a\right)}\left(\frac{1}{\rho_{2}^{\left(a\right)}}-\frac{1}{\rho_{2}^{\left(b\right)}}\right)=0,$$

where superscripts $\left(b\right)$ and $\left(a\right)$ denote the values before and after the relaxation step, respectively. Considering the five unknowns $e_{1}^{\left(a\right)},e_{2}^{\left(a\right)},\rho_{1}^{\left(a\right)},\rho_{2}^{\left(a\right)},p^{\left(a\right)}$, three more equations are needed to close the system. First, since ${\left(\alpha \rho \right)}_{k}$ is conserved during the relaxation, the saturation constraint $\sum_{k}\alpha_{k}=1$ reads:

(13) $$\frac{{\left(\alpha \rho \right)}_{1}^{\left(b\right)}}{\rho_{1}^{\left(a\right)}}+\frac{{\left(\alpha \rho \right)}_{2}^{\left(b\right)}}{\rho_{2}^{\left(a\right)}}=1\text{.}$$

The two more required equations are extracted from the phasic equations of state, which express the internal energy of the phase based on its pressure and density. This is straightforward when using simplistic equations of state, such as the ideal gas and stiffened gas EoSs described in A in their parametric forms, which read as:

(14) $$e_{1}^{\left(a\right)}=\frac{p^{\left(a\right)}+\gamma_{1}p_{\infty ,1}}{\left(\gamma_{1}-1\right)\rho_{1}^{\left(a\right)}},e_{2}^{\left(a\right)}=\frac{p^{\left(a\right)}+\gamma_{2}p_{\infty ,2}}{\left(\gamma_{2}-1\right)\rho_{2}^{\left(a\right)}}\text{.}$$

In this specific case, an analytical solution for the system of Eqs. (11), (12), (13), (14) exists, which is described in [45], [65]. However, in the case of complex equations of state, there is no analytical solution for this system. Therefore, an iterative solution should be tailored based on the particular formula of the utilised EoS. Herein, we present a general algorithm for this system based on tabulated data. In this regard, residuals associated with Eqs. (11), (12), (13) are defined:

(15) $${∊}_{1}=e_{1}^{\left(a\right)}-e_{1}^{\left(b\right)}-p^{\left(a\right)}\left(\frac{1}{\rho_{1}^{\left(a\right)}}-\frac{1}{\rho_{1}^{\left(b\right)}}\right),$$

(16) $${∊}_{2}=e_{2}^{\left(a\right)}-e_{2}^{\left(b\right)}-p^{\left(a\right)}\left(\frac{1}{\rho_{2}^{\left(a\right)}}-\frac{1}{\rho_{2}^{\left(b\right)}}\right),$$

(17) $${∊}_{3}=1-\left(\frac{{\left(\alpha \rho \right)}_{1}^{\left(b\right)}}{\rho_{1}^{\left(a\right)}}+\frac{{\left(\alpha \rho \right)}_{2}^{\left(b\right)}}{\rho_{2}^{\left(a\right)}}\right)\text{.}$$

This system can be solved iteratively using the multivariable Newton’s method. As the pressure and temperature are the inputs in the tabulated data, the system is solving for $T_{1}^{\left(a\right)},T_{2}^{\left(a\right)},p^{\left(a\right)}$ through the following steps:

1. The values of $T_{1}^{\left(a\right)},T_{2}^{\left(a\right)},p^{\left(a\right)}$ are initially guessed. For the first loop, the values from the previous time step are used.
2. Based on the guessed values, the corresponding densities and internal energies are interpolated from the table through bilinear interpolation. Therefore, the residual functions ${∊}_{1},{∊}_{2},{∊}_{3}$ are calculated.
3. The guessed values of step 1 are shifted by $\Delta T_{1}=\xi_{\left(T_{1}\right)}T_{1},\Delta T_{2}=\xi_{\left(T_{2}\right)}T_{2},\Delta p=\xi_{\left(p\right)}p$. Values used in this study are in the range of $10^{-3}<\xi_{\left(T_{1}\right)},\xi_{\left(T_{2}\right)},\xi_{(}p)<10^{-2}$. This allows to compute the partial derivatives of the residual functions with respect to temperature and pressure: $\frac{\Delta {∊}_{m,T_{1}}}{\Delta T_{1}},\frac{\Delta {∊}_{m,T_{2}}}{\Delta T_{2}},\frac{\Delta {∊}_{m,p}}{\Delta p}$, where *m* is the error index m=1,2,3:
  $$\begin{array}{c} \frac{\Delta {∊}_{m,T_{1}}}{\Delta T_{1}}=\frac{{∊}_{m}(T_{1}^{\left(a\right)},T_{2}^{\left(a\right)},p^{\left(a\right)})-{∊}_{m}(T_{1}^{\left(a\right)}+\Delta T_{1},T_{2}^{\left(a\right)},p^{\left(a\right)})}{\Delta T_{1}},\\ \frac{\Delta {∊}_{m,T_{2}}}{\Delta T_{2}}=\frac{{∊}_{m}(T_{1}^{\left(a\right)},T_{2}^{\left(a\right)},p^{\left(a\right)})-{∊}_{m}(T_{1}^{\left(a\right)},T_{2}^{\left(a\right)}+\Delta T_{2},p^{\left(a\right)})}{\Delta T_{2}},\\ \frac{\Delta {∊}_{m,p}}{\Delta p}=\frac{{∊}_{m}(T_{1}^{\left(a\right)},T_{2}^{\left(a\right)},p^{\left(a\right)})-{∊}_{m}(T_{1}^{\left(a\right)},T_{2}^{\left(a\right)},p^{\left(a\right)}+\Delta p)}{\Delta p}, \end{array}$$
4. The following iterative system in constructed based on the Newton’s method:
  (18) $${\left[\begin{array}{c} T_{1}\\ T_{2}\\ p \end{array}\right]}^{{\left(a\right)}^{\left(n+1\right)}}={\left[\begin{array}{c} T_{1}\\ T_{2}\\ p \end{array}\right]}^{{\left(a\right)}^{\left(n\right)}}-\left[J^{-1}{\left(T_{1},T_{2},p\right)}^{{\left(a\right)}^{\left(n\right)}}\right]{\left[\begin{array}{c} {∊}_{1}\\ {∊}_{2}\\ {∊}_{3} \end{array}\right]}^{{\left(a\right)}^{\left(n\right)}},$$
  where $J^{-1}$ stands for the inverse of the Jacobian matrix:
  $$J={\left[\begin{array}{ccc} \frac{\Delta {∊}_{1,T_{1}}}{\Delta T_{1}} & \frac{\Delta {∊}_{1,T_{2}}}{\Delta T_{2}} & \frac{\Delta {∊}_{1,p}}{\Delta p}\\ \frac{\Delta {∊}_{2,T_{1}}}{\Delta T_{1}} & \frac{\Delta {∊}_{2,T_{2}}}{\Delta T_{2}} & \frac{\Delta {∊}_{2,p}}{\Delta p}\\ \frac{\Delta {∊}_{3,T_{1}}}{\Delta T_{1}} & \frac{\Delta {∊}_{3,T_{2}}}{\Delta T_{2}} & \frac{\Delta {∊}_{3,p}}{\Delta p} \end{array}\right]}^{n}\text{.}$$
5. To increase the convergence speed and avoid instabilities, an under-relaxation coefficient $c_{\mathrm{urf}}=0.5$ was applied at each loop as:
  (19) $${\left[\begin{array}{c} T_{1}\\ T_{2}\\ p \end{array}\right]}^{{{\left(a\right)}^{\left(n+1\right)}}^{☆}}=\left(1-c_{\mathrm{urf}}\right){\left[\begin{array}{c} T_{1}\\ T_{2}\\ p \end{array}\right]}^{{\left(a\right)}^{n^{☆}}}+c_{\mathrm{urf}}{\left[\begin{array}{c} T_{1}\\ T_{2}\\ p \end{array}\right]}^{{\left(a\right)}^{\left(n+1\right)}},$$
  where superscript ${{\left(a\right)}^{\left(n+1\right)}}^{☆}$ denotes the updated values at the end of each iteration of the Newton’s method. The following criteria based on the change of the variables over the iterations is considered at each point:
  $$\left|\frac{\left({T_{1}}^{{\left(n+1\right)}^{☆}}-T_{1}^{☆}\right)}{T_{1}^{{\left(n+1\right)}^{☆}}}\right|+\left|\frac{\left({T_{2}}^{{\left(n+1\right)}^{☆}}-T_{2}^{☆}\right)}{T_{2}^{{\left(n+1\right)}^{☆}}}\right|+\left|\frac{\left(p^{{\left(n+1\right)}^{☆}}-p^{☆}\right)}{p^{{\left(n+1\right)}^{☆}}}\right|<\varepsilon ,$$
  where $\varepsilon =10^{-2}$ is sufficient for convergence to be achieved.
6. Calculating the equilibrium pressure and the corresponding phasic densities, the volume fractions can be updated as ${\left(\alpha \rho \right)}_{k}$ is conserved for phase *k*. From the relaxation step, the phasic densities and volume fractions are estimated properly as inferred from the numerical tests of [66].

### 3.3 Re-initialisation

After the hyperbolic step, the non-conservative internal energies do not infer a common relaxation pressure. In addition, the phasic internal energies, solved in a non-conservative manner, do not necessarily correspond to the total energy. Thus, a re-initialisation step is introduced to ensure that the non-conservative internal energies are consistent with a common equilibrium pressure and correspond to the total energy. This is summarized in the following steps:

1. The mixture internal energy $e_{m}$ is extracted from the total and kinetic energy computed in the hyperbolic step:
  (20) $$e_{m}=E-\frac{1}{2}\left(u^{2}+w^{2}\right)\text{.}$$
2. The mixture rule for the internal energies is considered as:
  (21) $$e_{m}=Y_{1}e_{1}+Y_{2}e_{2},$$
  in which the left hand side $e_{m}$ is known from the previous step while $Y_{1}$ and $Y_{2}$ are available from the relaxation step. Therefore, with the substitution of $e_{1}$ and $e_{2}$ with the corresponding pressure and densities based on the equations of state, the pressure will be the only unknown. In the case of the stiffened gas EoS, Eq. A.1, we obtain the following equation:
  (22) $$p=\;\frac{\rho \left(E-\frac{1}{2}\left(u^{2}+w^{2}\right)\right)-\left(\frac{\alpha_{1}\gamma_{1}p_{\infty ,1}}{\gamma_{1}-1}+\frac{\alpha_{2}\gamma_{2}p_{\infty ,2}}{\gamma_{2}-1}\right)}{\frac{\alpha_{1}}{\gamma_{1}-1}+\frac{\alpha_{2}}{\gamma_{2}-1}}\text{.}$$
  For more complex equations of state, however, there is no analytical solution of Eq. 21. Similar to generalisation made in the relaxation system, we present an iterative method valid for any arbitrary equation of state. In this regard, the residual based on Eq. 20 is considered:
  (23) $$∊=e_{m}-E_{m}+\frac{1}{2}\left(u^{2}+w^{2}\right)\text{.}$$
  Newton’s method for pressure reads:
  (24) $$p^{\left(n+1\right)}=p^{n}-{\left(\frac{∊}{{∊}_{p}^{\prime }}\right)}^{n},$$
  where ${∊}_{p}^{\prime }$ is the partial derivative of the residual function with respect to the pressure estimated as:
  (25) $${∊}_{p}^{\prime }=\frac{\Delta ∊}{\Delta p}=\frac{e_{m}\left(p+\Delta p\right)-e_{m}\left(p\right)}{\Delta p},$$
  where Δp represents a small change in pressure and can be estimated based on the pressure from the previous loop $\Delta p=\xi_{p}p$ for which $\xi_{p}=10^{-3}$ is recommended. Moreover, the initial guess values are considered based on the relaxation step. Similar to the relaxation step, an under-relaxation treatment is also considered to ensure stability. The pressure is computed when the solution converges $\left|\frac{\left(p^{\left(n+1\right)}-p^{n}\right)}{p^{\left(n+1\right)}}\right|<\varepsilon$; a suggested value of $\varepsilon =10^{-3}$ has been used.
3. Finally, the internal energies are recomputed with the pressure obtained in step 2. It is now ensured that the updated internal energies are in agreement with the total energy conservation.

## 4 Results and discussion

In this part, three spherical collapse cases with different initial pressure ratios are first presented, followed by the non-spherical collapse of a bubble near a rigid wall excited by a pressure pulse corresponding to conditions similar to those generated by commercial lithotripter ultrasound systems. Moreover, the effect of the distance between the bubble and the rigid wall is considered. A cavitation case is also presented in D as a benchmark test. In all simulation, the gas phase is assumed to be non-condensable and the effects of viscosity, heat conductivity, surface tension, and phase transition are neglected.

Each phase contains a small volume fraction of the opposite phase $\alpha_{\min}=10^{-6}$ in the initial setups to ensure the hyperbolicity of the system, as recommended in [45]. Moreover, the monotonized central slop limiter is used for the MUSCL reconstruction scheme explained in [45]. The time step is varying based on the CFL number which is set to 0.5.

The first case considered here is the symmetric collapse of an isolated air bubble with initial radius $R_{0}=1$ mm submerged in infinite water at rest; the different investigated are indicated in Table 1. This test is performed in 1D spherical coordinates. Initially, the pressure inside the air bubble $p_{\mathrm{air}}$ is uniform in $r=\left[0,R_{0}\right]$ while the surrounding pressure in $\left(r=\left(R_{0},L_{D}\right]\right)$ increases gradually towards the far-field $p_{f}$ [1]:

(26) $$p_{\mathrm{water}}\left(r\right)=p_{f}+\;\frac{R_{0}}{r}\left(p_{\mathrm{air}}-p_{f}\right),$$

where $L_{D}$ indicates the domain size. The grid cells are uniformly distributed in two regions with different resolutions. In the first region $r=\left[0,3R_{0}\right]$ containing the bubble and its neighbourhood, $3N_{R0}$ cells are uniformly placed where $N_{R0}$ denotes the number of cells per initial radius $R_{0}$. The number of $N_{R0}=100$ is sufficient to obtain the converged solution based on the grid resolution analysis provided in E. In the second region $r=\left(\left(3R_{0},L_{D}\right]\right)$, $N_{L}$ cells are used with the uniform distribution. The size of the domain $L_{D}$ is considered $20R_{0},50R_{0},80R_{0}$ with $N_{L}=2N_{R0},6N_{R0},10N_{R0}$ cells for case 1, 2, and 3, respectively, to be large enough to avoid any possible interaction between the wave reflection from the far-field and the bubble. Reflective and transmissive boundary conditions [60] are used for the bubble centre and the far-field region, respectively. Results obtained with the ideal and real gas equations of state are also compared to those obtained with the Keller-Miksis model. It should be mentioned that the Helmholtz EoS failed in the simulation of case 1 as the bubble properties in this case exceed the valid range of the Helmholtz EoS described in Section 2. To make the comparisons more clear, we use initial radius and Rayleigh collapse time to non-dimensionalise the radius and time respectively as follows [33]:

(27) $$R^{*}=\frac{R}{R_{0}},$$

(28) $$t^{*}=\frac{t}{0.915R_{0}\sqrt{\frac{\rho_{\mathrm{water}}}{p_{f}}}}\text{.}$$

**Table 1:** Initial conditions for the spherical bubble collapse cases.

	Variable					
Case	$p_{\mathrm{air}}$ (Pa)	$p_{f}$ (Pa)	$\rho_{\mathrm{air}}$ (kg/m^3^)	$\rho_{\mathrm{water}}$ (kg/m^3^)		
1	$1.01325\times 10^{5}$	$3.57589\times 10^{7}$	1.225	$9.982\times 10^{2}$		
2	$1.01325\times 10^{5}$	$3.57589\times 10^{6}$	1.225	$9.982\times 10^{2}$		
3	$1.01325\times 10^{5}$	$7.15178\times 10^{5}$	1.225	$9.982\times 10^{2}$		

**Fig. 2:**
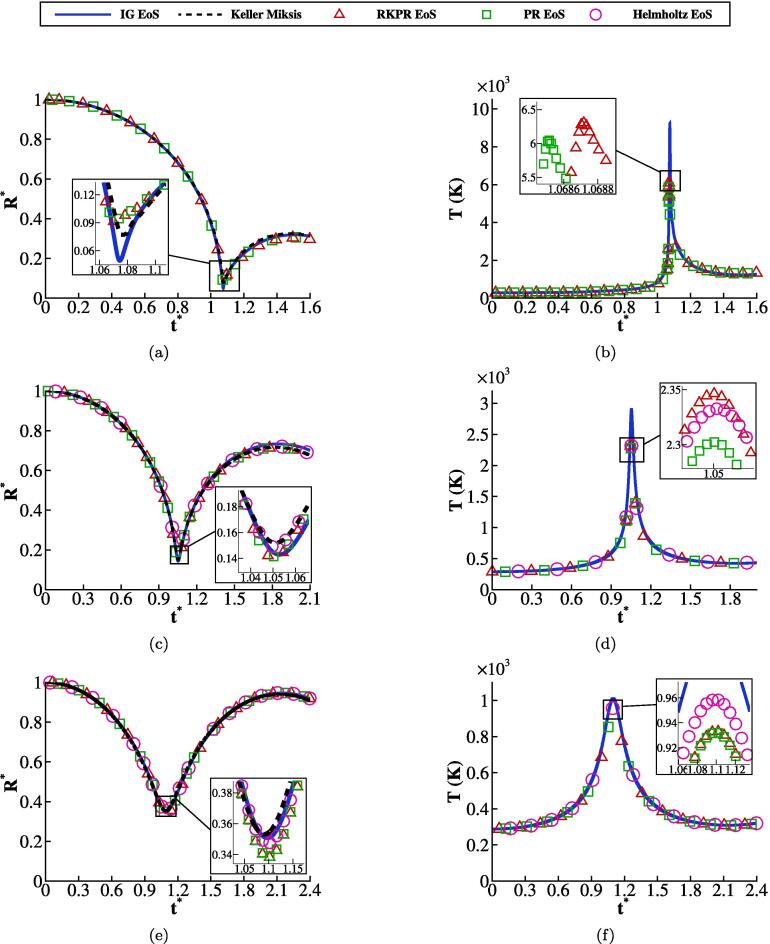
(a), (c), (e) Bubble dynamics and (b), (d), (f) space-averaged temperature obtained with different EoSs for collapse with initial pressure ratios of case 1, 2, and 3 in the first, second, and third rows, respectively.

As can be seen in Fig. 2, Fig. 2, Fig. 2e depicting cases 1, 2 and 3 respectively, the bubble undergoes a compression, the rate of which depends on the initial pressure ratio, followed by a rebound. The comparison with the Keller-Miksis model shows that the present method captures the compression and expansion rate with satisfactory accuracy for all cases. The temporal change of the space-averaged gas temperature inside the bubble is plotted for all cases in Fig. 2, Fig. 2, Fig. 2f using both the real and ideal gas EoSs. It is found that the predicted temperatures obtained with the three real gas EoSs are very similar, as expected considering the results presented previously in Fig. 1. On the other hand, the difference between the temperatures predicted by the real gas EoSs and ideal gas EoS is significantly affected by the initial pressure ratio. It is observed that for the violent collapse, case 1, the space-averaged temperatures obtained with the real-gas EoSs are ≈33% lower than the value predicted by the ideal gas EoS. The difference is negligible, however, in case 3 where the collapse is mild. The maximum temperature achieved during bubble collapse is reported for all cases investigated in Table 2.

**Table 2:** Maximum collapse temperature averaged in the space of the bubble interior for different initial pressure ratios with ideal and real gas EoS.

	EoS					
Case	IG	RKPR	PR	Helmholtz		
1	9230 (K)	6300 (K)	6050 (K)	-		
2	2900 (K)	2340 (K)	2300 (K)	2330 (K)		
3	1010 (K)	930 (K)	930 (K)	960 (K)		

**Fig. 3:**
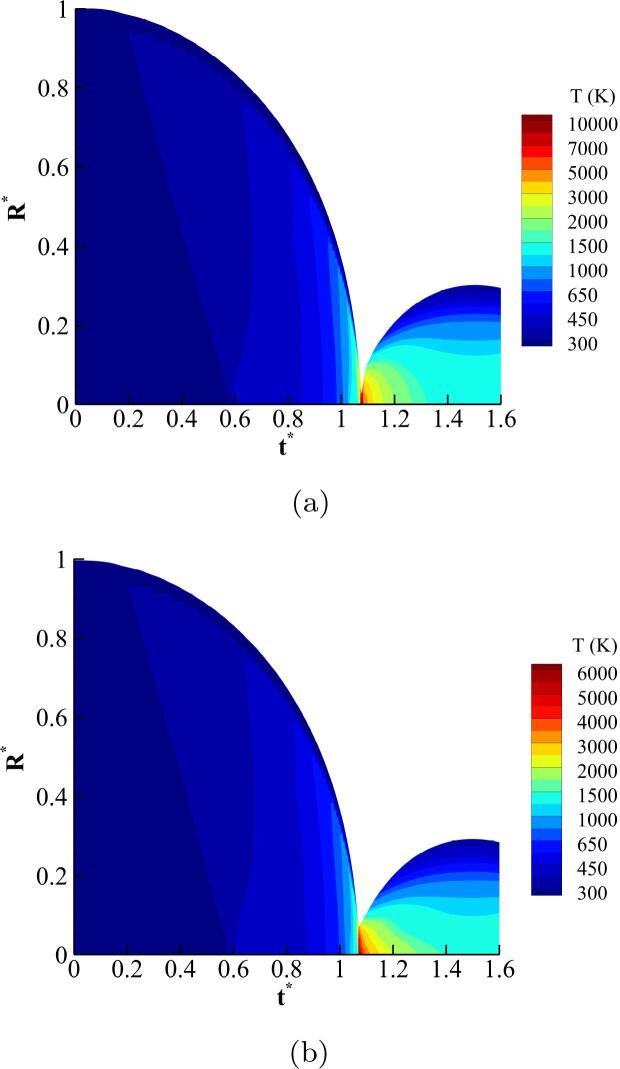
Spatio-temporal contours of temperature change during the bubble collapse obtained with: (a) IG and (b) RKPR EoSs.

To get more insight into the bubble spatial temperature variation, the spatio-temporal distribution of the gas temperature in $r=\left[0,R_{0}\right]$ obtained with the ideal gas and RKPR EoSs for case 1 is depicted in Fig. 3. In this figure, the vertical axis shows the non-dimensional space inside the bubble while the horizontal axis denotes the non-dimensional time. This representation in useful to illustrate the temperature locally inside the bubble during the entire simulation. It is evident that the bubble collapse undergoes a nearly isothermal process in the initial stage due to the slow collapse rate, followed by adiabatic heating. As the bubble approaches to its minimum size during the collapse, the temperature rises due to the very high compression rate and reaches a local maximum values of ≈10,000 K and ≈6,000 K predicted by the ideal gas and the RKPR EoS respectively. Subsequently, the bubble cools down during the expansion phase where gradients of temperature are observed.

**Fig. 4:**
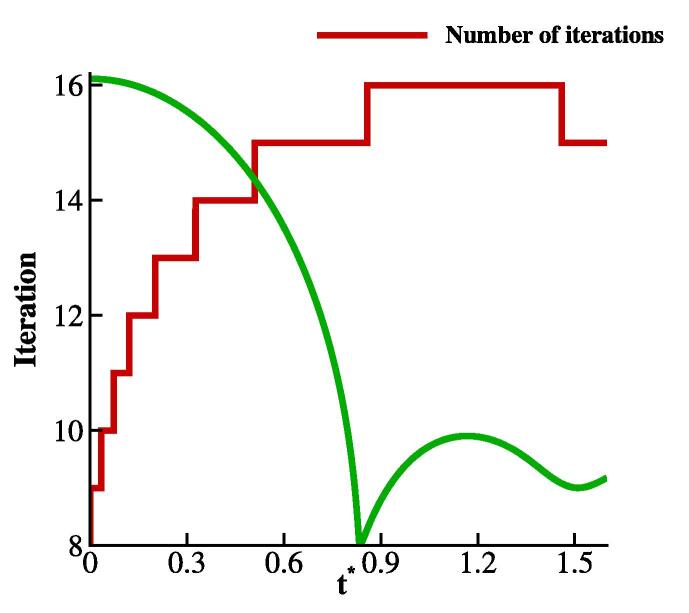
Space-averaged number of required iterations in the relaxation step for case 1 simulated with RKPR EoS. The bubble radius change over time (green line) is also plotted to observe the collapse stage.

It should be noted that the number of iterations required for the relaxation to converge varies in time. To demonstrate this, the average of this value in the whole domain has been reported in Fig. 4 for case 1 with the RKPR EoS. Accordingly, the required number of iterations reaches its maximum during the collapse to reach the convergence. The CPU time of the serial computation for this simulation is 320.85 s on an Intel Core i7-8850U CPU @1.8 GHz.

### 4.1 Shock-induced non-spherical collapse close to a rigid wall

Moving towards a case with more practical interest, a shock-induced bubble collapse near a rigid wall surface is examined. The bubble-wall arrangement resembles that of a lithotripter system. This test case was first introduced in the work of [50], where they studied the wall pressure subjected to the bubble collapse. In this setup, infinite impedance for the kidney stone is assumed to avoid any wave absorption in the boundary. It was shown that the wall pressure reaches values of GPa depending on the initial stand-off distance as well as the pulse width and amplitude. Herein, the focus is on the collapse temperature with the ideal and RKPR EoSs. Moreover, the wall pressure is depicted for various initial stand-off distances.

**Fig. 5:**
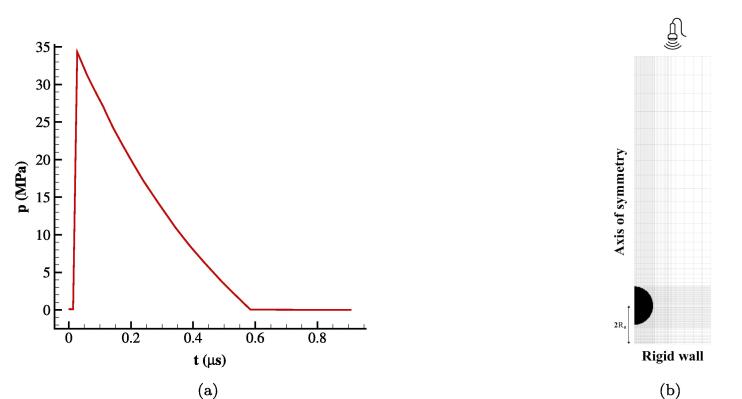
(a) Pressure pulse of the lithotripter and (b) schematic of the setup.

The compressive shock front from the upper boundary demonstrated in Fig. 5a represents the lithotripter pulse without the tensile part propagating in time; this is based on an analytical function described in [50]. Initially, the pressure is atmospheric in the whole domain and the water and air densities are $\rho_{\mathrm{water}}=998.2$ kg/m^3^ and $\rho_{\mathrm{air}}=1.125$ kg/m^3^, respectively. To reduce the computational cost, the case is simulated in 2D axisymmetric coordinates instead of the full 3D configuration. A schematic of the geometry and the mesh is presented in Fig. 5b. Different resolutions in $r=\left[0,1.2R_{0}\right],r=\left(\left(1.2R_{0},2R_{0}\right]\right)$, and $r=\left(\left(2R_{0},4R_{0}\right]\right)$ are used in the r-direction with the total number of $N_{r}=400$ cells. In the z-direction, however, the grid is uniform in $z=\left[0,1.2R_{0}\right]$ while the Vinokur function [67] is used in $z=\left(\left(1.2R_{0},15R_{0}\right]\right)$ for grid stretching with the total number of $N_{z}=750$ cells. The grid independence study is provided in E, showing that considering $N_{R0}=150$ cells in the initial setup is sufficient to obtain grid independent solutions. The reflective boundary condition is used for the axis of symmetry whereas for the right side and the bottom wall, the non-reflective and no-slip boundary conditions have been used, respectively. The bubble has an initial radius of $R_{0}=0.05$ mm while the initial stand-off distance case is $2R_{0}$.

**Fig. 6:**
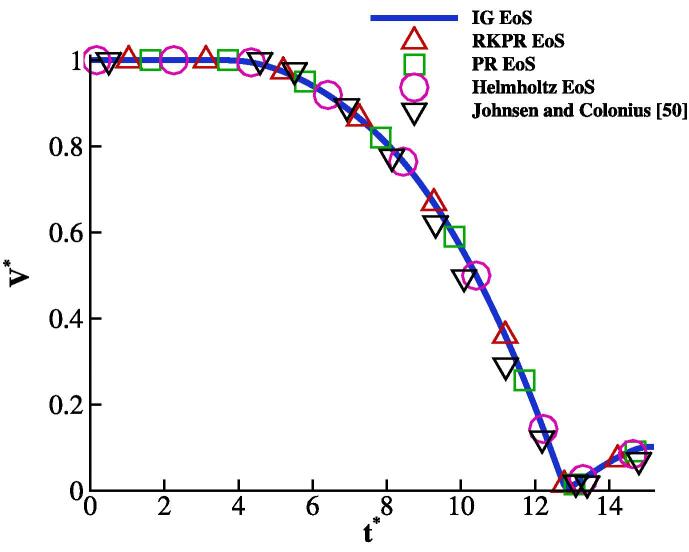
Bubble dynamics of shock-induced collapsing bubble compared with [50].

To be consistent with the reference [50], the variation of the bubble volume normalised with its initial value $V^{*}=V/V_{0}$ over non-dimensional time $t^{*}={\mathrm{tc}}_{L}/R_{0}$ is plotted in Fig. 6 where $c_{L}=1647$ m/s is the reference speed of sound. The results obtained with the ideal and real gas EoSs are compared with the study of [50]; overall, good agreement is achieved. It can be seen that the dynamics of the bubble is not affected by the gas thermodynamics. On the other hand, it was seen in the spherical bubble collapse cases that the bubble temperature predicted by the three real gas EoSs was nearly the same as shown in Fig. 2. Therefore, the RKPR EoS is used as the real gas EoS for the rest of the results.

**Fig. 7:**
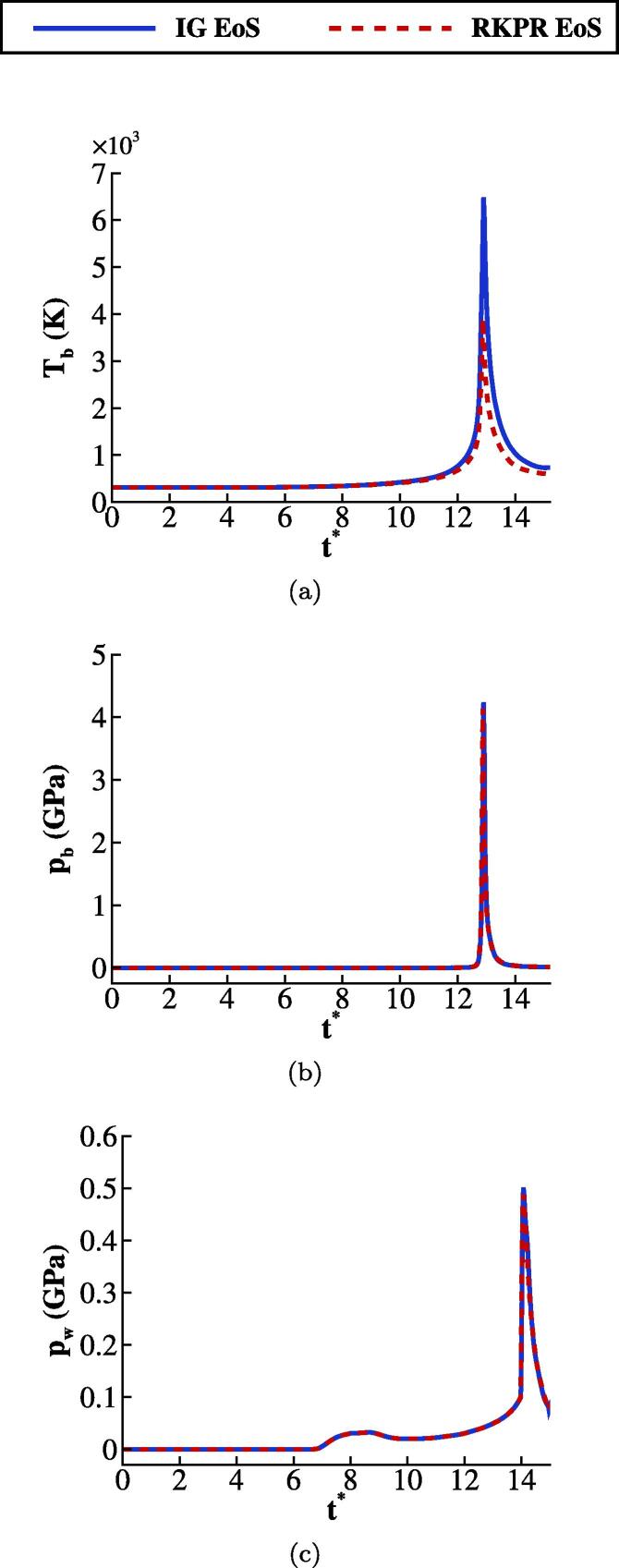
(a) and (b) Effect of the gas EoS on the space-averaged bubble temperature and pressure respectively, and (c) on the wall pressure for $H_{0}=2R_{0}$.

In Fig. 7a, it is observed that the bubble compression results to bubble space-averaged temperatures up to 6,500 K when the ideal gas EoS is utilised. The temperature predicted with the RKPR EoS is approximately 3,800 K which is ≈41% lower than the ideal gas EoS prediction. This noticeable temperature difference at $p_{b}\approx 4$ GPa, which is corresponding to the compression ratio of $4\times 10^{4}$, is in agreement with the comparison made in Section 2, Fig. 1 where ≈35% temperature difference between the ideal and real gas prediction for an isentropic compression at similar compression ratio was observed. Fig. 7b, however, shows a less significant difference in the bubble pressure which is only ≈2.3% based on our data. Similarly, in Fig. 7c, it is observed that the predicted wall pressure averaged in $r=\left[0,R_{0}\right]$ is less affected by the gas EoS due to the sufficiently large stand-off distance.

**Fig. 8:**
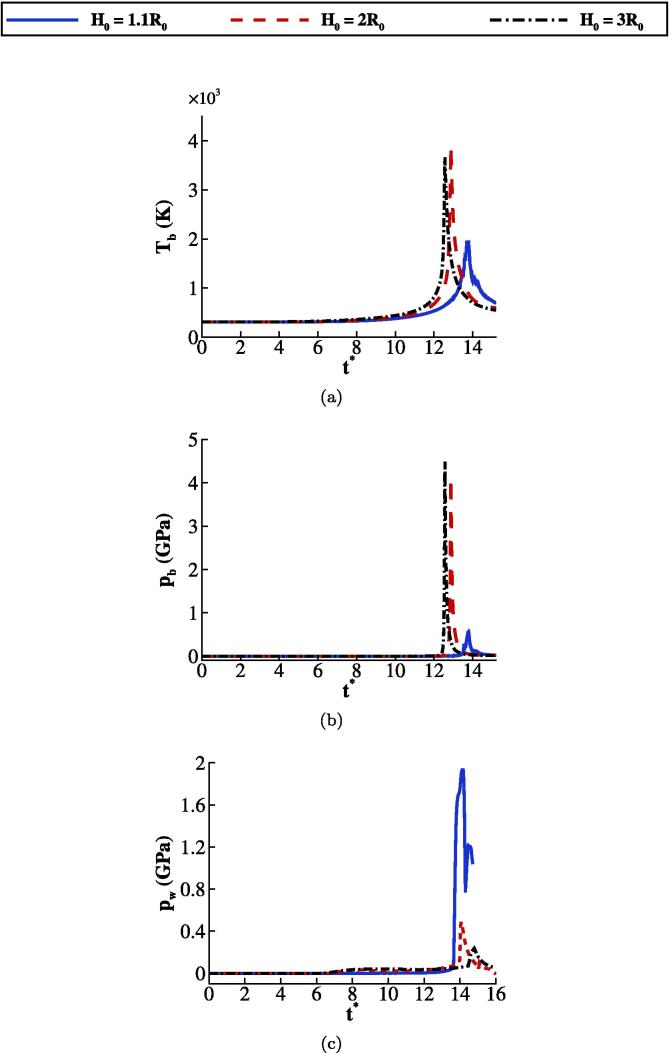
(a) and (b) Effect of the stand-off distance on the space-averaged bubble temperature and pressure respectively, and (c) on the wall pressure where RKPR EoS is used.

Simulations performed with the RKPR EoS for different stand-off distances namely $H_{0}=1.1R_{0},H_{0}=2R_{0}$, and $H_{0}=3R_{0}$ as shown in Fig. 8, Fig. 8c. It is observed that when the stand-off distance is minimum, i.e., $H_{0}=1.1R_{0}$, the collapse forms in a more asymmetric shape compared to the two other cases. Therefore, a less amount of energy can be concentrated inside the bubble. As a result, the maximum bubble temperature and pressure are lower when $H_{0}=1.1R_{0}$. On the other hand, the wall pressure peak, averaged in $z=\left[0,R_{0}\right]$, is the highest in this case as the shock immediately hits the wall.

**Fig. 9:**
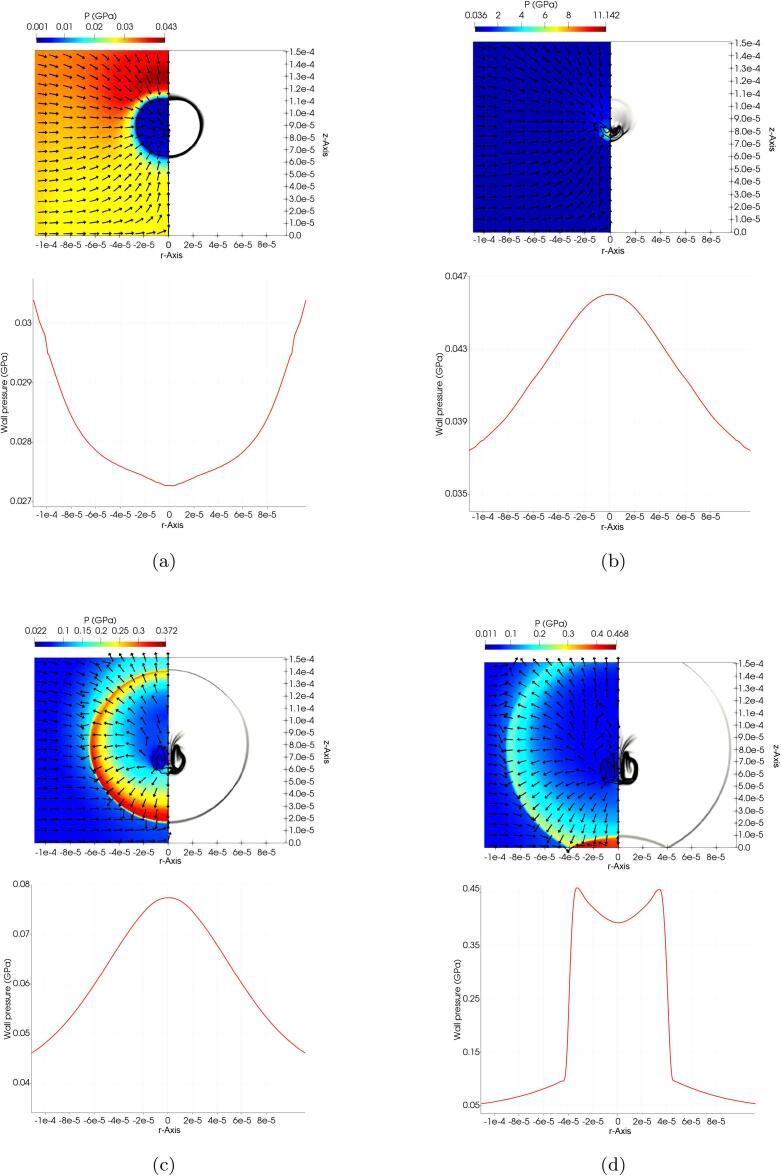
Pressure variation and velocity vectors (left half), numerical Schlieren (right left), and wall pressure over time in the 2D axisymmetric simulation of the non-spherical shock-induced collapse with the real gas EoS for $H_{0}=2R_{0}$. a) $t^{*}\approx 11.54$, b) $t^{*}\approx 12.82$, c) $t^{*}\approx 13.65$, d) $t^{*}\approx 14.11$.

Numerical Schlieren [68] is used as a useful gradient-based function for visualisation of the formed waves as well as the interface location [35], [69]:

(29) $$\phi =\exp\left(-\frac{\psi |\nabla \rho |}{\max|\nabla \rho |}\right),$$

where ψ is a scaling parameter to improve the visibility of the waves for which a value of 50 is used in the simulations. In the contour plots of Fig. 9, the temporal evolution of the pressure field *p* and velocity vectors (on the left-half), and the numerical Schlieren (on the right-half) are illustrated at the selected times, namely a) $t^{*}\approx 11.54$, b) $t^{*}\approx 12.82$, c) $t^{*}\approx 13.65$, d) $t^{*}\approx 14.11$ corresponding to different collapse stages. Moreover, the line plots of Fig. 9 represent the wall pressure $p_{w}$ at each time. The simulations are obtained for $H_{0}=2R_{0}$ with the RKPR EoS while no substantial difference for this set of variables were observed in the case of the ideal gas EoS. It can be seen that the emitted pressure pulse from the lithotripter hits the bubble at the top boundary creating a reflected rarefaction wave and a transmitted shock wave. The induced pressure gradient onsets the bubble collapse, as seen in Fig. 9a ($t^{*}\approx 11.54$). The transmitted wave is then reflected from the rigid wall and hits back the bubble from the lower boundary, compressing it further. At this stage, the peak pressure of the wall is ≈0.055 GPa. The liquid jet formation is evident in Fig. 9b ($t^{*}\approx 12.82$) as well as an emitted shock wave due to bubble collapse. Thirdly, the bubble expands into a toroidal-like shape and the emitted shock wave from the collapse travels toward the rigid wall, as seen in Fig. 9c ($t^{*}\approx 13.65$). Up to this point, the wall pressure peak is still less than 0.08 GPa. Lastly, the bubble expands further and the shock wave hits the rigid wall and abruptly increases its pressure to the peak value of above 0.45 GPa, as depicted in Fig. 9d ($t^{*}\approx 14.11$). This shows that the peak value of the wall pressure is strongly influenced by the collapse shock wave. Also, observing the wall pressure above 0.37 GPa in |r|<30 μm it seems that the wall can retain the high pressure value as the shock is passing outward from the centre.

**Fig. 10:**
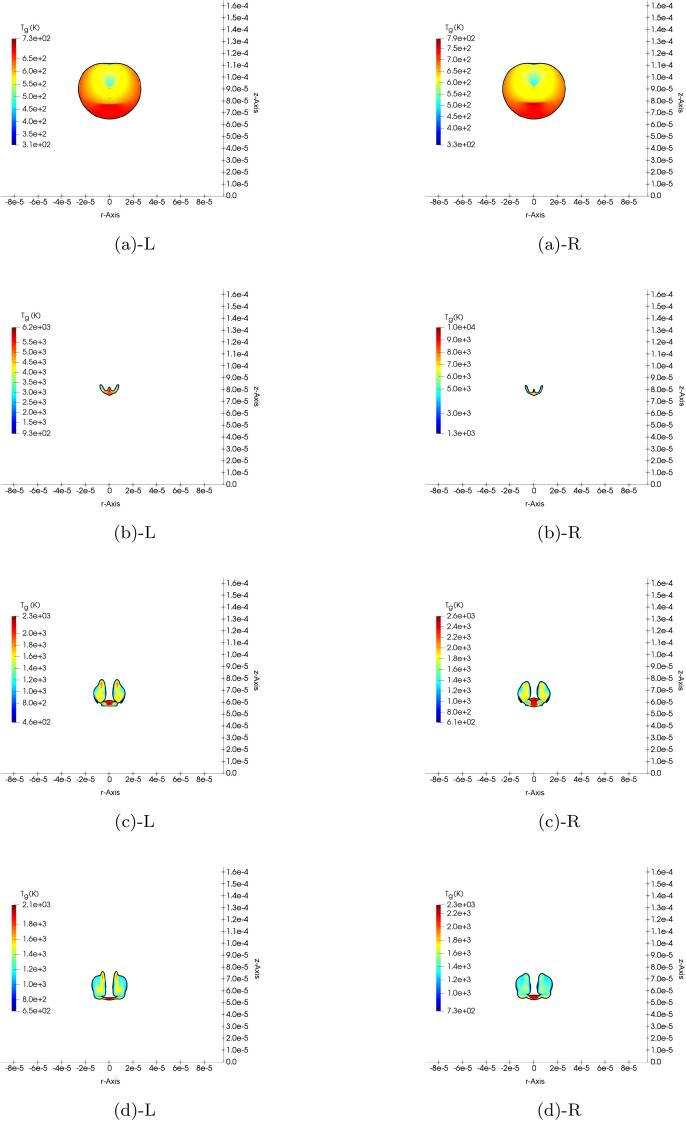
Gas temperature variation over time in the 2D axisymmetric simulation of the non-spherical shock-induced collapse with the real gas (left column) and ideal gas (right column) EoS for $H_{0}=2R_{0}$. a) $t^{*}\approx 11.54$, b) $t^{*}\approx 12.82$, c) $t^{*}\approx 13.65$, d) $t^{*}\approx 14.11$.

While the spatial average of the gas temperature presented in 7 is appropriate to monitor the bubble temperature over time, we are able to extract more insight about the temperature distribution and the real gas effects locally at different collapse stages with a 2D representation. Therefore, the evolution of the gas temperature is shown in Fig. 10 at the same selected times considered for Fig. 9, i.e., $t^{*}\approx 11.54$, b) $t^{*}\approx 12.82$, c) $t^{*}\approx 13.65$, d) $t^{*}\approx 14.11$. The results obtained with the RKPR EoS (left column) are compared with those with the ideal gas EoS (right column). In both cases, the temperature distribution inside the bubble is inhomogeneous as expected in non-spherical collapse. This temperature first increases due to the adiabatic during the compression phase. At this stage, the difference between the predictions by the ideal and RKPR EoSs is minor, with the maximum of ≈60 K in the spot shown in Figs. 10a ($t^{*}\approx 11.54$). However, this difference becomes substantial at the moment of collapse. At the selected $t^{*}\approx 12.82$, which is very close to the collapse moment, the maximum temperature predicted by the ideal gas EoS reaches ≈10000 K in the very small red spot in the centre as shown in Fig. 10b R which makes a difference of ≈3800 K with the maximum prediction by the RKPR EoS in Fig. 10bL. Although this maximum temperature difference happens only in a very small spot, the RKPR EoS predicts lower collapse temperature even in the surrounding of the spot. As the bubble expands, cooling of the gaseous content is expected. This is clearly seen in Figs. 10c ($t^{*}\approx 13.65$) and 10d ($t^{*}\approx 14.11$) with the maximum temperature difference of ≈300 K and ≈200 K, respectively.

## 5 Conclusion

A numerical model extending the work of [45] to account real-fluid EoS via tabulated data has been developed and applied to gas collapse cases. Both spherical and non-spherical collapse cases have been considered and compared with simulations obtained with the ideal gas EoS. The real gas effects become more dominant when the collapse is more violent, leading to a ≈33% difference (corresponding to ≈ 3050 K) in the space-averaged spherical collapse temperature. Also, a difference of ≈41% (corresponding to ≈ 2700 K) is observed for the space-averaged non-spherical collapse temperature. It is also shown that the difference is even more evident if the local extreme collapse temperature is considered. Therefore, it is concluded that the ideal gas assumption fails for temperature prediction regardless of the collapse sphericity.

## CRediT authorship contribution statement

**Saeed Bidi:** Conceptualization, Methodology, Software, Validation, Formal analysis, Investigation, Data curation, Writing - original draft, Writing - review & editing, Visualization. **Phoevos Koukouvinis:** Conceptualization, Methodology, Software, Validation, Formal analysis, Data curation, Writing - review & editing, Supervision. **Andreas Papoutsakis:** Writing - review & editing, Supervision. **Armand Shams:** Conceptualization, Methodology. **Manolis Gavaises:** Conceptualization, Resources, Writing - review & editing, Supervision, Project administration, Funding acquisition.

## Data access statement

The Supporting Information is available free of charge on the ACS Publications website at DOI: 10.25383/city.21262635

## Declaration of Competing Interest

The authors declare that they have no known competing financial interests or personal relationships that could have appeared to influence the work reported in this paper.

## Figures and Tables

**Table A.3:** Parameters for the cubic equations of state.

	EoS	
Parameter	PR	RKPR
u	1	$\delta_{1}+\delta_{2}$
w	2	$\delta_{1}\delta_{2}$
$\delta_{1}$	$1+\sqrt{2}$	$d_{1}+d_{2}{\left(d_{3}-1.168Z_{c}\right)}^{d_{4}}+d_{5}{\left(d_{3}-1.168Z_{c}\right)}^{d_{6}}$
$\delta_{2}$	$1-\sqrt{2}$	$\frac{1-\delta_{1}}{1+\delta_{1}}$
a	$0.45724\left(\frac{R_{u}^{2}T_{c}^{2}}{p_{c}}\right)$	$\frac{3y^{2}+3\mathrm{yd}+d^{2}+d-1}{{\left(3y+d-1\right)}^{2}}\left(\frac{R_{u}^{2}T_{c}^{2}}{p_{c}}\right)$
b	$0.07780\left(\frac{R_{u}T_{c}}{p_{c}}\right)$	$\frac{1}{3y+d-1}\left(\frac{R_{u}T_{c}}{p_{c}}\right)$
η	${\left[1+S\left(1-\sqrt{\frac{T}{T_{c}}}\right)\right]}^{2}$	${\left(\frac{3}{2+\frac{T}{T_{c}}}\right)}^{k}$
$\frac{d\eta }{\mathrm{dT}}$	$-\frac{S}{{\mathrm{TT}}_{c}}\left[1+S\left(1-\sqrt{\frac{T}{T_{c}}}\right)\right]$	$-\frac{3^{k}k}{T_{c}{\left(2+\frac{T}{T_{c}}\right)}^{k+1}}$
$\frac{d^{2}\eta }{{\mathrm{dT}}^{2}}$	$\frac{S^{2}}{2{\mathrm{TT}}_{c}}+\frac{S}{2\sqrt{T^{3}T_{c}}}\left[1+S\left(1-\sqrt{\frac{T}{T_{c}}}\right)\right]$	$\frac{3^{k}k\left(k+1\right)}{T_{c}^{2}{\left(2+\frac{T}{T_{c}}\right)}^{k+2}}$

**Fig. 11:**
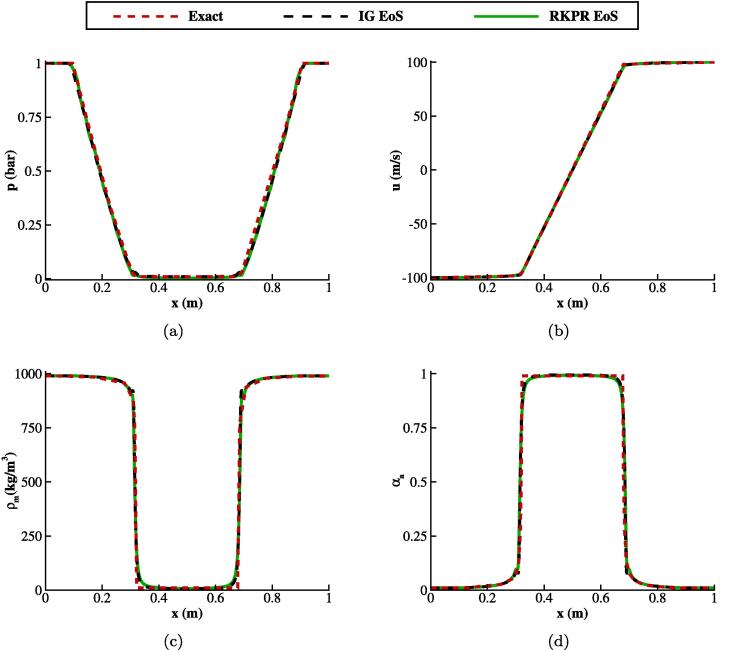
Numerical simulation of cavitation test with the ideal and real gas EoSs compared with the exact solution [45] a.t t=1.85 ms.

**Fig. 12:**
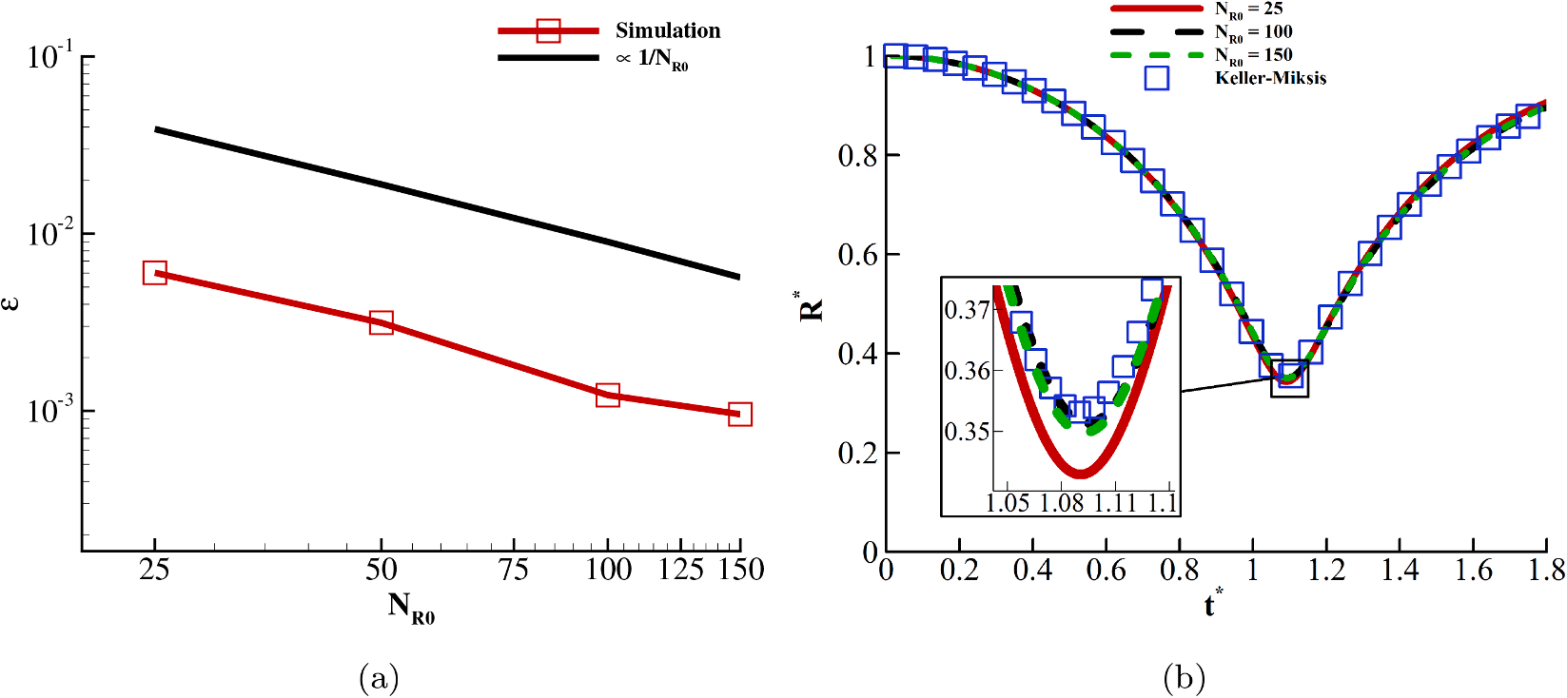
(a) Spatial convergence error. (b) Results with different grid resolutions for the spherical collapse case 3.

**Fig. 13:**
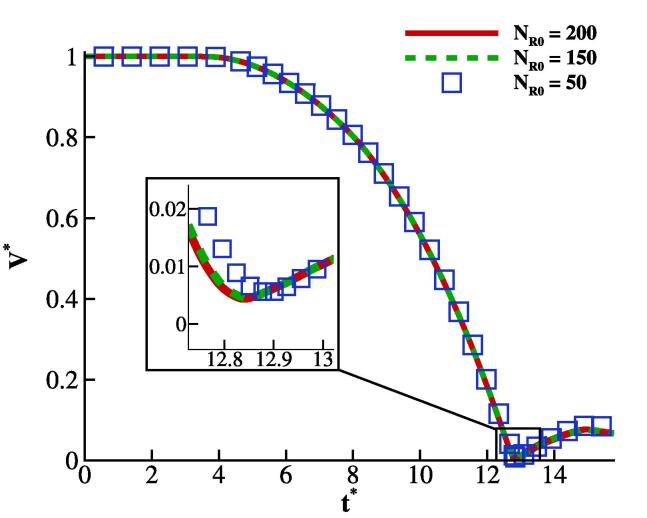
Grid analysis of the shock-induced non-spherical collapse close to a rigid wall.

## Appendix A Equations of state

### A.1 Stiffened gas for liquid

The stiffened gas EoS [49] is a simplified form of the Mie-Gr”uneisen EOS in which the pressure is defined as:

(A.1) $$p=\left(\gamma -1\right)\rho e-\gamma p_{\infty },$$

(A.2) $$T=\frac{p+p_{\infty }}{c_{v}\left(\gamma -1\right)\rho },$$

where *p* and *e* are the pressure and the specific internal energy, respectively. This EoS has been widely used in the literature, e.g. [32], [45], [56], [55], due to its simplicity. The parameters are set to $\gamma =\frac{c_{p}}{c_{v}}=4.4$ and $p_{\infty }=6\times 10^{8}$ Pa for water. With a thermal equilibrium assumption in the initial condition, the specific heat is computed which is used as constant without any physical meaning. It is noted that the value of $p_{\infty }=0$ and γ=1.4 converts it to ideal gas.

### A.2 Higher order equations of state for gas

Whereas ideal gas equation of state has proven quite successful in modelling gaseous behaviour, generalisation and expansion such a simple model for liquids and/or phase change was not straightforward. Even if simplistic extensions of the ideal gas or polytropic equation of state could cover for liquid behaviour (see for example the stiffened gas EoS above, or the Tait EoS for weakly compressible liquids respectively [70], these could not describe the thermodynamics of phase change. Considering the variation of pressure and specific volume along a constant temperature, it becomes apparent that to describe both liquid and vapor phases at the same time an equation of state of at least third order in respect to density is needed. Indeed, the first such equation of state was the Van der Waals EoS [71], which, in its derivation, included both repulsive forces between molecules (similar to the ideal gas EoS), but also the attractive intermolecular forces present in liquids. Despite the fact that the Van der Waals EoS could describe the critical point of matter and provide a theoretical insight in non-ideal effects, such as the Joule–Thomson coefficient, it proved quite inaccurate in predicting accurately the liquid vapor equilibrium [72], hence this model was surpassed by more advanced variations.

#### A.2.1 Peng-Robinson EoS

One of the most notable improvements of the Van der Waals EoS and a characteristic example of the cubic EoS family, is the Peng-Robinson EoS, which is commonly used for describing real fluid effects, phase change and transcritical/supercritical mixing, see e.g. [46], [73], [74]. Despite its success and its widespread role in chemical engineering, it suffers from deficiencies when predicting saturated liquid densities and speed of sound. Its formulation is discussed in the next section, as it is a subset of a generalised form to be discussed.

#### A.2.2 Redlich-Kwong-Peng-Robinson EoS

Cismondi et al. [47] combined the PR EoS and RK EoS to introduce the generalised RKPR EoS. In this equation, the density dependence of each earlier EoS is connected to improve modelling of the real fluid behaviour. The third parameter is used to make interpolations and extrapolations along the RK EoS and PR EoS. The analytical equations of the cubic EoSs are very similar. The general form of a cubic EoS is described as:

(A.3) $$p=\frac{\rho R_{u}T}{M_{w}-b\rho }-\frac{a\eta \left(T\right)\rho^{2}}{\left(M_{w}+\delta_{1}b\rho \right)\left(M_{w}+\delta_{2}b\rho \right)},$$

in which $R_{u}=8.3145\left(\frac{J}{\mathrm{mol}\;K}\right)$ is the universal gas constant and $M_{w}$ is the molecular weight of the phase. The attractive and repulsive molecular forces are represented by parameters *a* and *b* which are empirically determined functions of the critical temperature and pressure. The attractive parameter *a* is multiplied by a correction factor $\eta \left(T\right)$ to consider the species polarity. This factor is a correlation of acentric factor ω and the temperature. The acentric factor is derived either from experimental data or from accurate formulas as suggested in [75].The third parameter $\delta_{1}$ in the generalised RKPR makes interpolations and extrapolations along the RK EoS and PR EoS. This parameter depends on the critical compressibility factor of the fluid $Z_{c}$. Nevertheless, the value of $\delta_{1}=1+\sqrt{2}$ gives the PR EoS as a special case of the RKPR EoS. The parameters of the cubic equations of state are given in A.3. Thermodynamic relation for the internal energy in the RKPR EoS reads:

(A.4) $$\begin{array}{cc} e\left(T,\rho \right) & =e_{0}\left(T\right)+\int_{\rho_{0}}^{\rho }{\left[\frac{p}{\rho^{2}}-\frac{T}{\rho^{2}}{\left(\frac{\partial p}{\partial T}\right)}_{\rho }\right]}_{T}d\rho \\ & =e_{0}\left(T\right)+\frac{1}{\left(\delta_{1}-\delta_{2}\right){\mathrm{bM}}_{w}}\left[T\left(\frac{\partial a\eta }{\partial T}\right)-a\eta \right]\ln\left(\frac{M_{w}+\delta_{1}b\rho }{M_{w}+\delta_{2}b\rho }\right), \end{array}$$

in which subscript 0 denotes the ideal state.

The following parameters are used in Table A.3:

$$\begin{array}{cc} & d=\frac{1+\delta_{1}^{2}}{1+\delta_{1}},\\ & y=1+{\left[2\left(1+\delta_{1}\right)\right]}^{\frac{1}{3}}+{\left(\frac{4}{1+\delta_{1}}\right)}^{\frac{1}{3}},\\ & S=0.37464+1.54226\omega -0.26992\omega^{2},\\ & k=\left(1.168Z_{c}A_{1}+A_{0}\right)\omega^{2}+\left(1.168Z_{c}{B_{1}}_{+}B_{0}\right)\omega +\left(1.168Z_{c}C_{1}+C_{0}\right)\text{.} \end{array}$$

The specific heat capacities at constant volume and pressure are computed:

(A.5) $$c_{v}={\left(\frac{\partial e}{\partial T}\right)}_{\rho }=c_{v,0}+\frac{T}{\left(\delta_{1}-\delta_{2}\right){\mathrm{bM}}_{w}}\left(\frac{\partial^{2}a\eta }{\partial T^{2}}\right)\ln\left(\frac{M_{w}+\delta_{1}b\rho }{M_{w}+\delta_{2}b\rho }\right),$$

(A.6) $$c_{p}=c_{v}+\frac{T}{\rho^{2}}\frac{{\left(\frac{\partial p}{\partial T}\right)}_{\rho }^{2}}{{\left(\frac{\partial p}{\partial \rho }\right)}_{T}}\text{.}$$

Moreover, the speed of sound can be derived as:

(A.7) $$c^{2}={\left(\frac{\partial p}{\partial \rho }\right)}_{s}=\frac{c_{p}}{c_{v}}{\left(\frac{\partial p}{\partial \rho }\right)}_{T},$$

where s stands for the specific entropy.

#### A.2.3 c) Helmholtz energy EoS

The Helmholtz energy EoS is based on an ideal and residual Helmholtz energy formulation, where the residual term $\alpha^{r}$ is a large polynomial/exponential function, calibrated with experimental data [76]:

(A.8) $$\alpha \left(\delta ,\tau \right)=\frac{a\left(\rho ,T\right)}{\mathrm{RT}}=\alpha^{0}\left(\delta ,\tau \right)+\alpha^{r}\left(\delta ,\tau \right),$$

in which α is the non-dimensional Helmholtz energy and superscripts 0 and r represent the ideal gas and residual contributions, respectively. Moreover, $\delta =\frac{\rho }{\rho_{j}}$ is the reduced density and $\tau =\frac{T_{j}}{T}$ is the reciprocal reduced temperature where subscript j denotes the value at the maxcondentherm. The residual Helmholtz energy contribution can be computed as:

(A.9) $$\alpha^{r}\left(\delta ,\tau \right)=\overset{10}{\underset{k=1}{\sum }}N_{k}\delta^{i_{k}}\tau^{j_{k}}+\overset{19}{\underset{k=11}{\sum }}N_{k}\delta^{i_{k}}\tau^{j_{k}}e^{\left(-\delta^{l_{k}}\right)},$$

for which the coefficients $N_{k}$ and values of $i_{k},j_{k}$, and $j_{k}$ can be found in [76] as well as the ideal gas contribution.

## Appendix B Relaxation system

The relaxation step contains the following equations [45]:

(B.1) $$\frac{\partial \alpha_{1}}{\partial t}=\mu \left(p_{1}-p_{2}\right),$$

(B.2) $$\frac{\partial \alpha_{1}\rho_{1}e_{1}}{\partial t}=-p_{I}\mu \left(p_{1}-p_{2}\right),$$

(B.3) $$\frac{\partial \alpha_{2}\rho_{2}e_{2}}{\partial t}=p_{I}\mu \left(p_{1}-p_{2}\right),$$

(B.4) $$\frac{\partial \alpha_{1}\rho_{1}}{\partial t}=0,$$

(B.5) $$\frac{\partial \alpha_{2}\rho_{2}}{\partial t}=0,$$

(B.6) $$\frac{\partial \rho u}{\partial t}=0,$$

(B.7) $$\frac{\partial \rho E}{\partial t}=0\text{.}$$

## Appendix C HLLC Riemann solver

The HLLC solver is being used extensively calculating the fluxes with high accuracy. This solver is recommended for the present method [45], [55], [65]. In this solver, the bounds for the minimum and maximum signal velocities present in the solution of the Riemann problem can be estimated as:

(C.1) $$S_{R}=\max\left(u_{L}+c_{L},u_{R}+c_{R}\right),$$

(C.2) $$S_{L}=\min\left(u_{L}-c_{L},u_{R}-c_{R}\right)\text{.}$$

where *c* stands for the mixture sound speed. In case of using the parametric SG equation of state, the sound speed for each phase *k* reads:

(C.3) $$c_{k}^{2}=\frac{\gamma_{k}\left(p_{k}+p_{\infty ,k}\right)}{\rho_{k}}\text{.}$$

When the tabulated equation of state is considered, the speed of sound is computed based on $p_{k}$ and $\rho_{k}$ through an interpolation using the tabulated data. The intermediate wave speed can be calculated using the HLL approximation using the mixture density and pressure:

(C.4) $$S_{M}=\frac{{\left(\rho u^{2}+p\right)}_{L}-{\left(\rho u^{2}+p\right)}_{R}-S_{L}{\left(\rho u\right)}_{L}+S_{R}{\left(\rho u\right)}_{R}}{{\left(\rho u\right)}_{L}-{\left(\rho u\right)}_{R}-S_{L}\rho_{L}+S_{R}\rho_{R}}\text{.}$$

Once the wave speeds are estimated, the following variables are obtained:

(C.5) $${\left(\alpha_{k}\rho_{k}\right)}_{R}^{*}={\left(\alpha_{k}\rho_{k}\right)}_{R}\frac{S_{R}-u_{R}}{S_{R}-S_{M}},$$

(C.6) $${\left(\alpha_{k}\rho_{k}\right)}_{L}^{*}={\left(\alpha_{k}\rho_{k}\right)}_{L}\frac{S_{L}-u_{L}}{S_{L}-S_{M}},$$

(C.7) $$p^{*}=p_{R}+\rho_{R}u_{R}\left(u_{R}-S_{R}\right)-\rho_{R}^{*}S_{M}\left(S_{M}-S_{R}\right),$$

(C.8) $$E_{R}^{*}=\frac{\rho_{R}E_{R}\left(u_{R}-S_{R}\right)+p_{R}u_{R}-p^{*}S_{M}}{\rho_{R}^{*}\left(S_{M}-S_{R}\right)},$$

(C.9) $$E_{L}^{*}=\frac{\rho_{L}E_{L}\left(u_{L}-S_{L}\right)+p_{L}u_{L}-p^{*}S_{M}}{\rho_{R}^{*}\left(S_{M}-S_{L}\right)},$$

in which the mixture density and the total energy are:

(C.10) $$\rho_{R}^{*}=\underset{k}{\sum }{\left(\alpha_{k}\rho_{k}\right)}_{R}^{*},$$

(C.11) $$E=Y_{1}e_{1}+Y_{2}e_{2}+\frac{1}{2}u^{2}\text{.}$$

In the absence of the relaxation effects, the volume fraction is constant along the fluid trajectories:

(C.12) $$\begin{array}{c} \alpha_{\mathrm{kR}}^{*}=\alpha_{\mathrm{kR}},\\ \alpha_{\mathrm{kL}}^{*}=\alpha_{\mathrm{kL}}\text{.} \end{array}$$

Since the volume fraction does not change across the left and right wave, the density reads:

(C.13) $$\rho_{\mathrm{kR}}^{*}=\rho_{\mathrm{kR}}\frac{u_{R}-S_{R}}{S_{M}-S_{R}}\text{.}$$

To compute the internal energy jumps the Hugoniot relation is required:

(C.14) $$e_{k}^{*}-e_{k}+\frac{p_{k}^{*}+p_{k}}{2}\left(\frac{1}{\rho_{k}^{*}}-\frac{1}{\rho_{k}}\right)=0\text{.}$$

If the parametric equations of state are considered, the internal energy can be expressed as a function of pressure and density:

(C.15) $$p_{k}^{*}=p_{k}^{*}\left(e_{k}^{*},\rho_{k}^{*}\right)\text{.}$$

Therefore, there will be only one unknown in the Eq. (C.14). In case of using the SG equation of state, for instance, using Eq. (A.1):

(C.16) $$p_{k}^{*}\left(\rho_{k}^{*}\right)=\left(p_{k}+p_{\infty ,k}\right)\frac{\left(\gamma_{k}-1\right)\rho_{k}-\left(\gamma_{k}+1\right)\rho_{k}^{*}}{\left(\gamma_{k}-1\right)\rho_{k}^{*}-\left(\gamma_{k}+1\right)\rho_{k}}-p_{\infty ,k}\text{.}$$

Subsequently, the internal energy $e_{k}^{*}$ can be obtained as a function of the pressure and density using the equation of state. However, the Hugoniot relation in Eq. (C.14) should be solved iteratively for energy and pressure in the case of using the tabulated EoS for the gas since there is no analytical expression relating the pressure and the internal energy. Therefore, the residual is defined based on Eq. (C.14) and the Newton method is used to minimise the error iteratively.

## Appendix D Benchmark tests

### D.1 Cavitation

Cavitating of a water–air mixture is simulated with the RKPR EoS and IG EoS and the results are compared with the exact solution. Initially, the pressure is p=1 bar everywhere in the domain x=[0,1] m while a velocity discontinuity is placed in the middle of the domain x=0.5 m. The flow velocity on the right side has u=100 m/s while it is set to u=-100 m/s on the left side. The tube is filled with the liquid water with ρ=1000 kg/m^3^ while there is a very small air volume fraction $\alpha_{a}=10^{-2}$ in the domain.

Existence of the gas with the left and right expansion waves from the centre generate vacuum in the middle of the domain where the liquid pressure drops, and a cavitation pocket is formed. Good agreement between the results with ideal gas EoS and the exact solution at t=1.85 ms from [45] is demonstrated in Fig. 11 in which the two interfaces are captured with a high accuracy with 1000 cells. It should be noted that real gas effects are minor in this regime of pressure. Therefore, it is observed that the results with RKPR EoS is similar to the results with the ideal gas EoS.

## Appendix E Grid convergence

The discrepancy between the numerical results and the Keller-Miksis solution for the low initial pressure ratio case can be served as the error when the grid resolution is analysed [33]. In this regard, the error is defined as the $L_{2}$ error for case 3:

(C.17) $$\varepsilon =\overset{N_{i}}{\underset{i=0}{\sum }}=\frac{\left|R\left(t_{i}\right)-R_{\mathrm{KM}}\left(t_{i}\right)\right|}{R_{\mathrm{KM}}\left(t_{i}\right)},$$

in which ε is the error computed in the time $\left[0,2t_{c}\right]$ in $N_{t}$ time steps where $t_{c}$ is the collapse time and $R\left(t_{i}\right)$ and $R_{\mathrm{KM}}\left(t_{i}\right)$ are the radius from the simulation and the Keller-Miksis model, respectively. The results shown in Fig. 12a represent a first order convergence which is the expected rate in the case of the discontinuous flows [60]. The grid resolution analysis for the same case is shown in Fig. 12b.

Grid convergence for the shock-induced non-spherical bubble collapse is also shown in Fig. 13.
